# Does Death of a Family Member Moderate the Relationship between Religious Attendance and Depressive Symptoms? The HUNT Study, Norway

**DOI:** 10.1155/2012/396347

**Published:** 2012-05-13

**Authors:** Torgeir Sørensen, Lars J. Danbolt, Jostein Holmen, Harold G. Koenig, Lars Lien

**Affiliations:** ^1^MF Norwegian School of Theology, P.O. Box 5144, Majorstuen, 0302 Oslo, Norway; ^2^Centre for Psychology of Religion, Innlandet Hospital Trust, Ottestad, Norway; ^3^HUNT Research Centre, Department of Public Health and General Practice, Faculty of Medicine, Norwegian University of Science and Technology, Trondheim, Norway; ^4^Duke University Medical Center, Durham, NC 27710, USA; ^5^King Abdulaziz University, Jeddah, Saudi Arabia; ^6^Department of Mental Health and Addiction, University of Oslo and Oslo University Hospital, Oslo, Norway

## Abstract

*Background*. The death of a family member is a stressful life event and can result in an increased level of depressive symptoms. Previous American research has shown inverse relationships between religious involvement and depression. European investigations are few and findings inconsistent; different contexts may have an important influence on findings. We therefore investigated the relationship between attendance at church/prayer house and depressive symptoms, and whether this relationship was moderated by the death of a close family member, in Norway. *Methods*. A population-based sample from the Nord-Trøndelag Health Study, Norway (HUNT 3, *N* = 37,981), was the population examined. Multiple regression and interaction tests were utilised. *Results*. Religious attendees had lower scores on depressive symptoms than non-attendees; death of a close family member moderated this relationship. The inverse relationships between attendance at church/prayer house and depressive symptoms were greater among those experiencing the death of an immediate family member in the last twelve months compared to those without such an experience, with men's decrease of depressive symptoms more pronounced than women's. *Conclusion*. In a population-based study in Norway, attendance at church/prayer house was associated with lower depressive symptoms, and the death of a close relative and gender moderated this relationship.

## 1. Introduction

Previous research has shown that loss of family members is associated with increased level of depression [1]. Religious involvement, in turn, has been associated with better psychological outcomes for people undergoing stressful life events [2]. However, even if people with religious or spiritual beliefs have better outcomes in response to negative life events in some studies [3], there is still much to be learned about how this relationship comes about [4].

Numerous studies from the USA have reported inverse relationships, both cross-sectional and longitudinal, between organizational religious involvement and depression [5, 6]. Among the most common measures used to assess religiosity in this field has been a single item, attendance at religious services [7].

The relationship between religious activity and depression in the European context is largely unexplored, and results of previous research are inconsistent. This may have to do with the different populations being studied in terms of the distribution of life stressors. In a study that examined religion and depression in eleven Western European countries, no direct association between religious indicators (including weekly church attendance) and depressive symptoms was found in this community-based sample [8]. Even though religious attendance has been associated with fewer depressive symptoms in some studies [9], stressful life events may affect this relationship. In fact, Braam [10] found that bereaved and nonmarried church attendees had slightly higher depression scores if they had high levels of orthodox belief.

Cultural context may be of importance when studying relationships between religion and mental health [11]. The inverse relationship between religiosity and depression appears stronger in countries with populations characterized by lower socioeconomic status (higher stress), high rates of religious attendance, and Catholic rather than Protestant majorities [6]. The present study is utilizing a large population-based sample from Norway consisting primarily of Protestants, mostly individuals from the middle class with low rates of attendance at church or prayer house. We examine here how the relationship between religious involvement and depressive symptoms operates in the presence or absence of the death of an immediate family member.

The aim of this study was therefor to explore (a) the relationship between attendance at church/prayer house and depressive symptoms and (b) whether a stressful life event, such as death of an immediate family member, moderates the relationship between attendance at church/prayer house and depressive symptoms in a Norwegian population.

## 2. Methods

Our data was collected in Nord-Trøndelag, a county in central Norway with a total population of 130,708 inhabitants [12]. With regard to characteristics such as geography, economy, industry, income, age distribution, types of illness, and mortality, this population is quite similar to the general Norwegian population [13]. However, education levels are lower than the national average, and the inhabitants live largely in rural districts and in small towns with up to 20,000 people.

The religious context is quite homogenous, since approximately 90% of the inhabitants belong to the Church of Norway (Lutheran). At the same time, frequency of religious attendance at churches or prayer houses is low compared to other countries, with 13% of the population attending once a month or more often, and only 3.6% attending weekly or more [14]. Furthermore, 47% seek God's help when they need strength and solace, and 75% state that the Christian worldview comes closest to their own.

### 2.1. Data Source

We utilized data from the third wave of the population-based Nord-Trøndelag Health Study (2006–2008), the HUNT 3 [15]. All inhabitants in Nord-Trøndelag aged 20 and over were invited to participate (*N* = 94,121). Of this group, 50,405 people completed Questionnaire 1 (Q1) and attended a screening visit, where clinical measures were administered and blood samples taken. Of these, 41,174 also completed Questionnaire 2 (Q2), received at the screening visit and returned it by mail. Within Q2, 37,981 participants responded to the question about attendance at church/prayer house, and this was our final sample. There were no substantial differences between the sample at Q1 and the final sample at Q2 regarding mean age (52.8 years versus 53.4 years) or gender (54.7% versus 55.9% women).

### 2.2. Measures

The *depressive symptoms* subscale of the Hospital Anxiety and Depression Rating Scale (HADS) administered at Q2 consisted of seven items [16]. The responses to each item ranged from 0 to 3 in severity. The subscale scores were constructed by adding the item scores, with the overall scores ranging from 0 to 20. Cronbach's alpha for the seven items in our sample was 0.75.

*Attendance at church or prayer house* was measured by the question, “How often in the last six months have you been to church or prayer house?” Responses were “never,” “1–6 times in the last 6 months,” “1–3 times/month,” and “more than 3 times/month.” This variable's applicability is evaluated [14], and it is found to measure attendance at religious service in addition to visits at funerals, weddings, baptisms, and concerts, as well as religious meetings in prayer houses for other reasons. For bivariate analyses this variable was dichotomised as “never” or “yes.”

*Death of an immediate family member* was measured by the question “Serious life events in the last twelve months: Has a member of your immediate family died?” (“No” and “Yes”). The dataset also included questions regarding relationship breakups and imminent mortal danger because of a serious accident, catastrophe, violent situation, or war. However, analyses investigating these two variables' interaction with attendance at church/prayer house, utilizing depression as dependent variable, showed no significant relationship. Consequently, they were not targeted for further investigation.

*Age* was calculated from the individual's date of birth and was analysed as a continuous variable. The participants' *education level* was obtained from Statistics Norway [12] and was dichotomised into lower (≤12 years) or higher (>12 years). *Relationship status *was dichotomised as single, separated, divorced, or widow(er) versus married/cohabiting.

### 2.3. Statistical Analyses

Frequency distributions were used to derive the characteristics of the sample. One-way ANOVA was utilized for bivariate analyses between attendance at church/prayer house and depressive symptoms stratified by the experience of death of an immediate family member. A multiple-regression model including all variables predicted depression scores for the entire sample. The same model was employed to test the interaction between attendance at church/prayer house and death of an immediate family member by including an interaction term in the model. Also, an interaction term of “Attendance at church or prayer house by Death of an immediate family member by Gender” was utilized to examine the effect of this three-way interaction. Demographics, socioeconomic characteristics and attendance at church/prayer house were included as independent variables in the regression analyses, together with death in family. The significance level was set at *P* < 0.05. SPSS Version 19 was used for all analyses [17].

The HUNT study is licenced by the Norwegian Data Inspectorate. Both the HUNT study and the research in this paper were approved by the Regional Committee for Medical and Health Research Ethics (REK) in Central Norway.

## 3. Results

The average score of depressive symptoms was 3.3 (women 3.1, men 3.6). 10.2% of the sample had experienced the death of an immediate family member (women 10.6%, men 9.7%). 40.8% never attended church or prayer house, 45.9% had attended one to six times over the last six months, 9.7% attended one to three times per month, and 3.6% more than three times per month. Mean age in the sample was 53.4 years, 29.2% had more than 12 years of education, and 60.9% lived in coupled relationships (Table 1).

In bivariate analysis, those who had experienced death within their immediate family had higher scores on depressive symptoms than those who had not (Table 2). Those who attended church or prayer house who had not experienced death in their immediate family had a lower depressive symptoms score, 3.18 (95% CI 3.14–3.22), than nonattendees, 3.35 (95% CI 3.30–3.40). Those attending church or prayer house who had experienced death in their immediate family also had lower depressive symptoms scores (3.70, 95% CI 3.58–3.82) compared to nonattendees (4.11, 95% CI 3.92–4.30) (Table 2).

Regression analysis controlling for all variables, including “Death of an immediate family member during the last 12 months,” revealed an inverse relationship between attendance at church/prayer house and depressive symptoms. Coefficients for the relationship between attendance and depressive symptoms were *B* = −0.18 (*P* = 0.03) for attendance >3x/month, *B* = −0.26 (*P* < 0.001) for 1–3x/month, and *B* = −0.25 for 1–6x/last 6 months (*P* < 0.001), compared to those who never attended church or prayer houses (Table 3(a)). In the relationship between the stressful life event of a death within one's immediate family and depressive symptoms the coefficient was *B* = +0.36 (*P* < 0.001) (Table 3(a)).

There was a very close to significant interaction between attendance at church/prayer house and the death of an immediate family member, with depressive symptoms as dependent variable (*P* = 0.053) (Table 3(b)). When investigating the depressive symptoms coefficients on attendance at church/prayer house, quite distinct differences between the “No” group and the “Yes” group with respect to the variable of “Death in immediate family” occurred (cf. annotation “c” and “d”). In this context, stratified analysis was appropriate.

Separate regression models were run with attendance at church/prayer house and other independent variables showing coefficients for depressive symptoms in the presence and absence of death of a family member (Table 4). The inverse relationships between attendance at church/prayer house and depressive symptoms were greater among those experiencing the death of an immediate family member in the last twelve months (never as reference: >3x/month *B* − 0.63, *P* = 0.01; 1–3x/month *B* − 0.25, *P* = 0.14; 1–6x/last 6 months *B* − 0.40, *P* = 0.001), compared to those without a death of an immediate family member (never as reference: >3x/month *B* − 0.11, *P* = 0.20; 1–3x/month *B* − 0.27, *P* < 0.001; 1–6x/last 6 months *B* − 0.24, <0.001) (Table 4).

A three-way interaction term (attendance at church/prayer-house by death of an immediate family member by gender) in the regression model was significant (*P* < 0.05). Regression models of the relationship between attendance at church or prayer house's and depressive symptoms were then rerun separately by death and no death of a close family member, and by gender (Table 5). The inverse relationship between attendance at church/prayer house and depressive symptoms was particularly strong among men with a death of an immediate family member, displayed with gradients, compared to women. In the group without a death in their immediate family during the last twelve months, depressive symptoms coefficients for men and women were at the same level, with the exception of men attending more than three times per month, who in fact had an increase of depressive symptoms (not significant) (Table 5).

## 4. Discussion

We found an inverse relationship between attendance at church/prayer house and depressive symptoms after adjustment for death in immediate family, age, gender, relationship status, and education. We also found that the death of an immediate family member within the last twelve months moderated the relationship between attendance at church/prayer house and depressive symptoms in such a way that the inverse relationship was even greater among those who had experienced a family death. Finally, among those experiencing the death of an immediate family member, the inverse relationship between attendance at church/prayer house and depressive symptoms appeared to be more pronounced among men.

Our study sample is characterized by considerable homogeneity, being primarily Protestant with low attendance at church/prayer house, and with individuals mostly from the middle class. Despite differences in cultural and religious contexts, the findings from this study are comparable to those reported in the USA [18]. However, among women in our sample without a death in immediate family the scores of depressive symptoms seem to be at the same level independent of high or low attendance frequency. On the other hand, a recent population-based American study reported lower depression scores the more the participants went to church [19].

Religiously involved people also had lower levels of depression in a European, Dutch sample [9]. Copland's study [8] including data from eleven different European countries (no Scandinavian countries) showed no direct association between religious indicators, for instance weekly service attendance and depression. Different measures of religious involvement and different samples regarding religious affiliation and context may partly explain the different findings, including ours.

The most frequent attendees are likely strongly affiliated to their religion. If this is correct, we found that strongly affiliated men who had experienced the death of an immediate family member over the last twelve months had a noticeably lower depressive symptoms score, with a coefficient of −0.89 (Table 5). This was also the case for the most frequent female attendees, but not as strong, with a coefficient of −0.49. This contrasts with other European findings where religiously affiliated people were more likely to experience depression than those not affiliated with religion when losing a child [20]. Also, bereaved people with strong orthodox beliefs had somewhat higher scores of depression [10]. This comparison may also indicate different religious contexts within the European environment, or different measures of religiosity. The Northern European folk-church religiosity, described by Davie as “belonging without believing” [21], may represent some differences compared to continental countries with high levels of orthodox beliefs or a high percentage of Catholics. Such beliefs may be more likely to be associated with rigid or guilt-producing religiosity [6].

In general, it is known that women are more religiously active than men [14]. From research it is also known that women often have greater health benefits from religious activity than men [5, 6, 22]. We found a significant inverse association between attendance at church/prayer house more than three times per month, and depression for women without a death in their immediate family in the last twelve months. The opposite occurred for men, as the depressive symptoms increased to some degree (cf. <3x/month), but not significantly (Table 5). On the other hand, among men who had experienced death in their immediate family, the lower scores on depressive symptoms were displayed by gradients and were remarkably lower on the values 1–3x/month and >3x/month compared to women. Thus, this association was more pronounced for men than women, even though the inverse relationship between religious attendance and depressive symptoms was greater for women with the death of an immediate family member, compared to those without such an experience. The difference may be considered notable due to the statistical significant three-way interaction between attendance in church/prayer house, death of a close family member, and gender. As this is a cross-sectional study, we can only propose possible interpretations of such a finding. However, it could be interesting to investigate further in a longitudinal study whether women benefit more from attendance at church/prayer house than men over the long term, whereas men may turn to their church only when they are under greater stress and need religion in order to cope.

The large population-based sample involving a religiously homogenous environment is an asset of the present study. Participants were asked a wide range of questions related to health and psychosocial issues without focusing on religion. Consequently, the risk of sample bias due to providing socially desirable responses is likely to be lower than in studies focused primarily on religion that are carried out in religious areas of the world.

A limitation of the study is its cross-sectional design without the possibility to determine causal effects and directions between variables. Use of only a single measure of religious involvement (attendance) also represents a limitation, since different measures of religion may generate different findings for the relationship between religion and depression [23]. Multiple-item measures could have other psychometric advantages. Nevertheless, the design of HUNT 3 precluded inclusion of multi-item measures of religious involvement, and the measure of attendance at church/prayer house employed here is widely used internationally as a measure of religiosity. In HUNT 3, attendance at church/prayer house is usually related to participation in the setting of the evangelical Lutheran Church of Norway. In addition to attendance at religious service, the current item also includes visits at funerals, weddings, baptisms, and concerts, as well as religious meetings in prayer houses for other reasons.

## 5. Conclusions

In this study we found that attendance at church/prayer house was significantly and inversely related to depressive symptoms. Death of an immediate family member interacted with attendance at religious services such that the inverse relationship between religious attendance and depressive symptoms was strongest among those who had lost an immediate family member in the past 12 months. This interaction was particularly strong in men. These results add to the growing literature base examining relationships between religious involvement and depression in European settings and are the first for Scandinavian countries examining how this relationship is moderated by a major stressful life event. Attendance at church/prayer house can be considered a means of coping [24]. These findings may contribute to awareness concerning patients' coping resources and suggest that attendance at church or prayer house may be a resource for stressed patients in healthcare settings [25, 26].

## Figures and Tables

**Table 1:** Participants of The HUNT Study (HUNT 3, 2006–08) answering questions about religious attendance. Characteristics of the sample.

	Total (*N* = 37,981)		Women (*N* = 21,247)		Men (*N* = 16,734)	
	Values (SD) range	*N*	Values (SD) range	*N*	Values (SD) range	*N*
Depression, mean (SD), Range	3.3 (2.9) 0–20	37,622	3.1 (2.9) 0–20	21,023	3.6 (2.9) 0–20	16,599
Death in immediate family (%)	10.2	3,755	10.6	2,182	9.7	1,573
Religious attendance (%)						
More than 3x/month	3.6	1,382	3.8	816	3.4	566
1–3x/month	9.7	3,671	10.4	2,205	8.8	1,466
1–6x/last 6 months	45.9	17,447	46.6	9,909	45.0	7,538
Never	40.8	15,481	39.1	8,317	42.8	7,164
Age, mean (SD), Range	53.4 (15.6) 20–96	37,981	52.5 (16.0) 20–96	21,247	54.4 (14.9) 20–96	16,734
Education (%)						
Up to 12 years	70.8	26,684	69.0	14,534	73.1	12,150
More than 12 years	29.2	11,014	31.0	6,537	26.9	4,477
Married/Couple (%)	60.9	23,092	57.8	12,259	64.8	10,833

**Table 2:** Religious attendance and depressive symptom scores measured by HADS, stratified by stressful life event (death in immediate family over the last 12 months).

		Mean	SD	95% CI	*P* values	*N*	Post hoc^b^
	Religious attendance (%)				<0.001		
	*A*: More than 3x/month	3.47	2.92	3.32–3.63		1,366	*A* > *D*
	*B*: 1–3x/month	3.57	2.86	3.47–3.66		3,639	*B* > *C*
	* C*: 1–6x/last 6 months	3.20	2.78	3.14–3.22		17,308	*C* < *B*
							*C* < *D*
	*D*: Never	3.43	3.06	3.39–3.48		15,309	*D* > *C*

Stratification:							
No death in immediate family	Religious attendance, Yes (%)^a^	3.18	2.76	3.14–3.22	<0.001	19,177	
	Religious attendance, No (%)	3.35	3.02	3.30–3.40		13,686	
					
Death in immediate family	Religious attendance, Yes (%)^a^	3.70	3.03	3.58–3.82	<0.001	2,459	
	Religious attendance, No (%)	4.11	3.39	3.92–4.30		1,256	

^a^“Yes” equals any religious attendance the last 6 months. “No” equals never attendance the last 6 months

^b^Significant differences (*P* < 0.05) by Tukey post hoc tests.

**Table:** (a) Regression model for entire sample

	Coefficients^a^	*P* values
Death in immediate family (ref. “No”)	0.36	<0.001
Religious attendance (ref. “Never”)		<0.001
>3x/month	−0.18	0.03
1–3x/month	−0.26	<0.001
1–6x/last 6 months	−0.25	<0.001
Age	0.03	<0.001
Women (ref. “men”)	−0.35	<0.001
Married/Couple (ref. “unmarried/not couple”)	−0.41	<0.001
Low education, ≤12 years (ref. “>12 years”)	0.58	<0.001
*R* ^2^	0.057	

**Table:** (b) Regression model including interaction term

	Coefficients^a,b^	*P* values
Death in immediate family (ref. “No”)^c^	0.49	<0.001
Religious attendance^d^		<0.001
>3x/month	−0.10	0.24
1–3x/month	−0.25	<0.001
1–6x/last 6 months	−0.24	<0.001
Religious attendance^e^		
>3x/month	−0.69	0.002
1–3x/month	−0.32	0.03
1–6x/last 6 months	−0.43	<0.001
*Interaction term*: “Religious attendance By Death in immediate family”		0.053
*R* ^2^	0.057	

^a^Unstandardized.

^b^Adjustment for age, sex, relationship status, and education.

^c^Difference in the “Never” group for religious attendance.

^d^Differences from the “Never” group for religious attendance, within the “No”-group for death in immediate family.

^e^Differences from the “Never” group for religious attendance, within the “Yes”-group for death in immediate family.

**Table 4:** Coefficients for the association between depressive symptoms and religious attendance, stratified by stressful life event (death in immediate family), controlling for age, gender, relationship status, and education.

	Coefficients^a^	*P* value
*Not experienced death in immediate family*		
Religious attendance^b^		<0.001
>3x/month	−0.11	0.20
1–3x/month	−0.27	<0.001
1–6x/last 6 months	−0.24	<0.001
*R* ^2^	0.056	

*Experienced death in immediate family*		
Religious attendance^b^		0.002
>3x/month	−0.63	0.01
1–3x/month	−0.25	0.14
1–6x/last 6 months	−0.40	0.001
*R* ^2^	0.043	

^a^Unstandardized.

^b^Reference group: “Never”.

**Table 5:** The association between depressive symptoms and religious attendance, stratified by stressful life event and gender, controlling for age, relationship status, and education.

	Religious attendance	Coefficients	*P* values
*Not experienced death in family *			
Women (Overall *P* < 0.001)	>3x/month	−0.25	0.03
	1–3x/month	−0.26	<0.001
	1–6x/last 6 months	−0.23	<0.001
*R* ^2^		0.054	
Men (Overall *P* < 0.001)	>3x/month	0.08	0.55
	1–3x/month	−0.27	0.003
	1–6x/last 6 months	−0.25	<0.001
*R* ^2^		0.047	

*Experienced death in family *			
Women (Overall *P* = 0.004)	>3x/month	−0.49	0.11
	1–3x/month	−0.06	0.77
	1-6x/last 6 months	−0.52	0.001
*R* ^2^		0.045	
Men (Overall *P* = 0.041)	>3x/month	−0.89	0.03
	1–3x/month	−0.59	0.03
	1–6x/last 6 months	−0.25	0.15
*R* ^2^		0.041	
